# Our Duty in Relation to Alcohol

**Published:** 1910-06

**Authors:** John M. Shaller

**Affiliations:** Denver, Colo.


### Our Duty in Relation to Alcohol.

BY JOHN M. SHALLER, M. D., DENVER, COLO.

Alcoholic drinks should never be prescribed without first taking into consideration their highly narcotic property. If it is desired to obtain results similar to those of opium, alcohol is the remedy to use.

After full physiological doses, both alcohol and opium first produce a "so-called" stimulating effect. This effect is probably more in the nature of delirium which many poisons produce. Excessive "so-called" stimulation, which follows larger doses of alcohol, shows more positively, by various strange antics, that it is not the result of pure heart stimulation. The increased flow of blood through the brain does not, of itself, produce cerebral excitement, resembling intoxication. Stimulation is accompanied with increased heart action, and should be accompanied by increased blood pressure. But as is well known, narcotics cause dilatation of the arterioles and flushing of the capillaries, conditions opposite to increased blood pressure.

The increase in pulse rate is not subjectively felt, but the effect of dilation is felt by a general feeling of warmth and flushing of the face. The head feels fuller, and a sense of general well-being is manifest. All of these sensations are produced through the action of the vaso-dilator nerves. The increased amount of blood in the skin produces there an apparent increase of heat. Extra heat is not created. The internal heat is conveyed in the larger amount of blood sent to the surface. Actual loss of heat, therefore, results from increased radiation. This loss is felt later by chilliness when this particular physiological effect has worn off and the vaso-constrictor nerves assume control.

Alcohol produces its effects rapidly. For example, when given for intestinal colic, by thus diverting the blood, through the action of the vaso-dilator nerves on the arterioles, the patient does not experience relief until the usually cold surface becomes warm.

The probabilities are that involuntary muscle fibres of the intestines and stomach are also relaxed at the same time as are the muscular coats of the blood-vessels.

When a hypodermic injection of morphia is given for colic, precisely similar results are produced. The patient nearly always remarks, "My skin is getting warm and I feel better."

It is for these primary physiological effects, which create a feeling of warmth and well-being, and for the relief of depression or some other feeling of discomfort that alcoholics are indulged in by those accustomed to taking them. We must fully realize, when prescribing alcoholics in slight or in chronic ailments, that they are as strongly narcotic as opium when given in equivalent, narcotic doses. We are particular about giving opium in slight or in chronic cases, and we should be just as particular about liquors, because the danger of producing habit is very great.

The matter of confidence in our patients in regard to their ability to control their appetites may be beautiful and complimentary, but it does not prevent the narcotic properties of alcohol from producing a habit, nor does it prevent its irritating property from increasing the connective tissue.

As physicians we can do a great deal toward lessening the greatest evil in existence. If we were to take a decided stand against it, we would do more good than all the temperance societies and churches combined. We are thoroughly familiar with post-mortem results, of hardened, hobnail livers, granular kidneys, edematous brains and ulcerated stomachs. We know all about the dropsical patients in hospitals, about the lost minds in insane asylums, about fearful hereditary effects of alcohol in the production of idiots, and about many other brain and nervous conditions. And yet we have all known of physicians who were familiar with all of these effects, and who saw them every day—even while they themselves were under the narcotic influence of alcohol.

It is really sad that our profession loses so many of its bright men to the powerful influence of narcotics. And how does this start? Frequently in attempts to be sociable, a good fellow, or for business reasons, or not to appear odd or a crank in the eyes of friends, probably also by taking whiskey to relieve pain or fatigue.

*Alcohol is a Narcotic.*—It has more victims than opium. Alcohol produces more marked pathological changes in all the organs than does opium. These changes alone should make strong impressions upon physicians as to the powerful irritating effects of alcohol.

Any substance that can change the structure of organs so that even with the microscope it is sometimes difficult to find normal tissue is not particularly safe to prescribe in chronic conditions.

And the pitiable mental consequence should prove effective warnings of its detrimental narcotic action upon the brain. But as physicians we are guardians—not of our own health only—nor of the health of our patients only—but also of that of the general public.

We are familiar with the beautiful and intricate arrangement of cells as they are aggregated and formed into complicated organs. We have, therefore, no right to delude ourselves, or permit others to be deluded, as to the consequences of drinking alcoholics for pleasure, because we know that alcohol utterly destroys the complex organism which started as a single cell and evolved into the complete mysterious structure of perfect man.

Hence, we should not use an irritant narcotic as a beverage or permit others to do so, since it produces an enormous increase of connective tissue, which so crowds upon the working cells, or produces fatty degeneration of cells, both of which conditions interfere with their functions, prevent their growth and later completely destroys them. If we ignorantly or willfully persist in destroying our own organs, we surely have no right to transmit such infirmities to children and make them bear the consequences of cell destruction. Nor should we permit others to do so. What kind of a paradox is that, of a man, who by profession has knowledge of these facts and changes, and then destroys his own organs, changes his own body into a walking pathological museum, and transmits these degenerate results to children. We prevent unhealthy animals from tarnsmitting their kind, but we do not interfere with men and women. We very kindly take care of their blasted progeny in prisons, almshouses and insane asylums. How much more human and humane if all this could be prevented.

In order to transmit the evil of alcoholism, it is not necessary that parents be chronic drunkards. If one or both parents are intoxicated at the time of conception, the child is likely to pay the penalty by some departure from health.

This one serious phase of temperance is not a Sunday school matter. It is not a fad, a sentiment, or a crankism. It is not a church or a W. C. T. U. question. It is one for trained medical men to consider seriously. The hereditary problem of intemperance needs the combined force and brains of the medical profession to handle it.

This profession has the requisite knowledge, the executive ability, and the power to do wonders in its effects to prevent man from destroying his mind and his body, and from transmitting mental, physical and moral weakness to his progeny.

The medical profession as a unit, if seriously and earnestly inclined, could put a decided check upon alcoholic evils. The question is—will the profession do it? I believe it will if it can be properly interested.

The chief conditions to be overcome are indifference and the erroneous beliefs which customs and teachings, both present and past, have fastened upon us. We all know their depressing influence, more or less. If these customs are evil ones, continuous association with them makes us indifferent to their evils, and we are even inclined to uphold and defend them. Sympathy for humanity, however, we have in abundance, and this is the call that will become an uplifting influence. Our business is to relieve suffering, and if there is suffering anywhere and of a preventable nature, it is surely where intemperance prevails.

For us the fight is not in any sense directed against the saloon, but it is for man himself and for his family.

Intemperance is found wherever there is moderate drinking, for it is the logical sequence. No temperate drinking—no intemperate drinking.

Temperate or moderate drinking is not so prevalent as in former years, and consequently intemperance is also lessened.

Many of the leaders of various professions are now temperance workers; this gives a certain positive standing and dignity which removes much of the derision that formerly fell to the early supporters of this question.

The physician should consider the scientific side only. That part which concerns the evil consequences that befall the entire race.

This should be done irrespective of any consequences that may result to those who oppose temperance movements.

The sole object should be to prevent sickness, immorality, crime, insanity and hereditary transmission.

As individuals each physician can do much, however, by discouraging moderate drinking, by not recommending the continuous and indiscriminate use of alcoholics as tonics, or appetizers, because they are narcotics.

If alcohol is recognized as a drug with narcotic, stimulating and irritating properties, and prescribed only when conditions indicate, just as with any other drug, the effect upon the public would be wonderful. There would be one less excuse to drink, and our profession would not be blamed for encouraging a custom that has lamentable consequences.

People can not live for themselves alone. Circumstances and the conduct in their lives influence their progeny for many generations to come. Therefore, they are culpable if they handicap children, and be the means of indefinitely transmitting mental, physical and moral obliquities. Authorities state that from 50 to 64 per cent of chronic drunkards have inebriate ancestry.

Is there any wonder that drunkards are morally degenerated, that they are mentally deluded, that they become liars, criminals and insane? How can it be otherwise with atrophy of cerebral cells, with effusions, and with increase of connective tissue?

No wonder that every function is interfered with, because the protoplasm, the cell substance itself, is the first to feel the paralyzing power of the narcotic and the irritant effects of alcohol.

With the marked changes produced in the brain cells, it is natural to expect brain and nervous diseases to be transmitted. The narcotic property of alcohol is the indirect cause of all organic changes produced through it, because it induces the habit. The irritating properties alone would not create the drug habit. In fact, alcoholics are taken only because they are narcotics.

One particular feature connected with drinking is that men seldom realize that they are under the influence of a narcotic. They either do not know, because they are narcotized, or they do not want to acknowledge it. While they should not be blamed for this latter, yet it would be better to recognize the narcotic effects early and make similar efforts to prevent and cure them, as in the case of opium. Besides, this narcotic effect should be more fully made known to the general public, and the doctors are best informed and best equipped to do so.

Men, generally, when they take wine or whiskey, do not know that they are taking chances with a narcotic, irritant poison. This is something with which no one can afford to take unnecessary risks. As physicians, in the treatment of mild or chronic diseases, we should not thoughtlessly compel our patients to take any chances of establishing a bad habit. In severe or in acute cases there are better remedies.

Unless something very radical interferes with the liquor business, the statistics show that each year will produce more drunkards, as well as more of all the varied evils that spring from drunkenness.

Who will fill the ranks? Why, you and I, and others who take alcoholics moderately as a beverage, and will not consider their strong, dominant, narcotic property. You and I, as physicians, may send some to that rank if we thoughtlessly prescribe alcoholics and overlook their narcotic effects. Let us be careful about this. Let us use all our influences. They are many and strong. It is easy for us to educate the general public along such lines. Let us lend our help to all movements which have as an end the prevention of evils. Let us spread the knowledge on every possible occasion by joining and supporting all medical movements that have for their object the study of the alcohol question.

Let us always be on the side which is striving to protect humanity, particularly to protect women and children from the many evils of intemperance.—*Medical Council,* Philadelphia.

---

"Growl, and the way looks dreary,
Laugh and the path is bright,
For a welcome smile
Brings sunshine, while
A frown shuts out the light.

"Sing, and the world's harmonious,
Grumble, and things go wrong;
And all the time
You are out of rhyme
With the busy bustling throng."

